# Expanding access for COVID-19 patients by transforming a burn unit into a closed-circuit unit for surgical patients: experience from an academic medical center in Jordan

**DOI:** 10.1186/s13037-020-00251-9

**Published:** 2020-06-05

**Authors:** Diab Bani Hani, Omar Altal, Abdelwahab Aleshawi, Ala”a Alhowary, Basil Obeidat

**Affiliations:** 1 Faculty of Medicine, Jordan University of Science and Technology, P. O. Box: 3030, Irbid, 22110 Jordan; 2 King Abdullah University Hospital, Irbid, 21110 Jordan

**Keywords:** COVID-19, Burn unit, Cross-infection

The current coronavirus disease 2019 (COVID-19) pandemic highlights the importance of a mindful utilization of financial and human resources. Preventing infections and preserving resources and manpower are crucial in healthcare. It is important to ensure the ability of the surgeons and the specialized interventionalists to function through this pandemic. A tremendous effort should be achieved to minimize cross-infections in this sector. In addition, more efforts should be conducted to monitor surgical patients diagnosed with COVID-19 because they have higher mortality rates. A study by Lei et al. was conducted retrospectively on 34 patients with COVID-19 who underwent surgery. They reported that all patients developed COVID-19 pneumonia shortly after surgery with abnormal findings on chest computed tomographic scans. Also, they reported that 15 (44.1%) patients required admission to the intensive care unit (ICU) during disease progression, and 7 patients (20.5%) died after admission to the ICU [1].

Another study was conducted in Italy regarding the protocols of dealing with operative patients in the era of COVID-19 pandemic. They recommended that the designated COVID-19 operating areas must be allocated to the operating room closest to the entrance of the theater block entrance [2]. Also, they advised that patients’ transit to and from the operating room must be as quick as possible and a pre-defined direct pathway must be kept as short as possible and away from other patients within the hospital in order to minimize the chances of cross-infection. In addition, they recommended to minimize the number of operators and other staff who should be equipped with personal protection equipment. At the end of the operation and during the recovery phase, the patient must be assisted directly in the operation room until ready to be transferred back to the inpatients place of stay. The time needed to return to wards must be reduced in order to minimize contact between COVID-19 positive patients and the surrounding environment [2]. The role of personal protection equipment was emphasized by Bianco et al. [3]. Another important point is how to stratify elective surgery during the pandemic. Stahel recommended that elective operations can be stratified into “essential“, which implies that there is an increased risk of adverse outcomes by delaying surgical care, versus “non-essential “or “discretionary“, which implies that there is no increased risk [4]. Moreover, Chadi et al. hypothesized that the minimal-invasive surgery has more advantages in a safe and controlled fashion [5]. However, they recommended ultimately to proceed with technical approaches they are comfortable with to ensure no added risk to the patient and operating room team occur [5].

Theoretically and practically, the aforementioned protocols carry a risk for hospital facilities contamination, and healthcare staff and other admitted patients cross-infection. Patients with COVID-19 are transferred in an “open circuit” even with the maximal strict adherence to the protocols [6, 7]. In addition, these protocols may affect the clinical status of these patients who; as reported by Lei et at; are at increased risk of postoperative complications and death [1]. Accordingly, at King Abdullah University Hospital in Jordan, we have prepared a “close circuit” model for surgical patients diagnosed with COVID-19. This “close circuit” model is being the burn unit at our center. The burn unit is equipped with 10 rooms. Each room in the burn unit has a bed in negative pressure environment. Also, each room can be adjusted to become an intensive-like care unit with intensive monitoring devices and mechanical ventilation. The rooms can be considered as a recovery room for each surgical patient. Moreover, the burn unit at has an operating theater that was supplied with the required surgical and interventional devices for all specialties and with the required anesthetic equipment (3 kits for general anesthesia, 3 kits for spinal anesthesia, 3 kits for epidural anesthesia and 3 kits for local anesthesia). Additionally, a neonatal coat and portable neonatal incubator were supplied. This unit is located above the emergency department and has its own elevator. All entrances were locked except for the main entrance which has double electronic doors. The changing rooms and the shower bath are located between the double doors. Furthermore, the healthcare staff including nurses, anesthesiologists and surgeons were supplied with the necessary personal protection equipment and includes: filtering face piece facial mask, disposable long sleeve waterproof coats and gowns, disposable double pair of nitrile gloves, protective goggles, disposable head caps, disposable long shoe covers and alcohol hygiene. The healthcare employees reside in this unit in an arranged schedule. After the completion of the shifts, the healthcare employees were isolated for 14 days in special comfortable isolated facilities without contacting anyone and they offered the COVID-19 tests before the end of the isolation. Successfully, two cesarean sections by spinal anesthesia were performed for two pregnant ladies. The neonates were admitted in another prepared rooms in the burn unit. It is important to highlight that the instruments from this unit have their own releasing track that is controlled by the infection control team. Also, the operations for non-COVID-19 patients were ceased except for the emergency or “essential” cases. Fortunately, and due to the reduced social activity by the quarantine, no burn cases were reported from all Northern of Jordan; which is cover by our center. However, the center has an intermediate care unit which was prepared for any burn case. Now, and after more than 1 month of the first reported case of COVID-19 in Jordan, the number of new cases is zero in Northern of Jordan. Among the surgical patient, no death was encountered and all patients achieved the recovery from the virus. Most importantly, we did not report any cross-infection in the healthcare employees and the other admitted seronegative patient. Figure 1 can demonstrates the blueprint for the burn unit at our center.

**Fig. 1:**
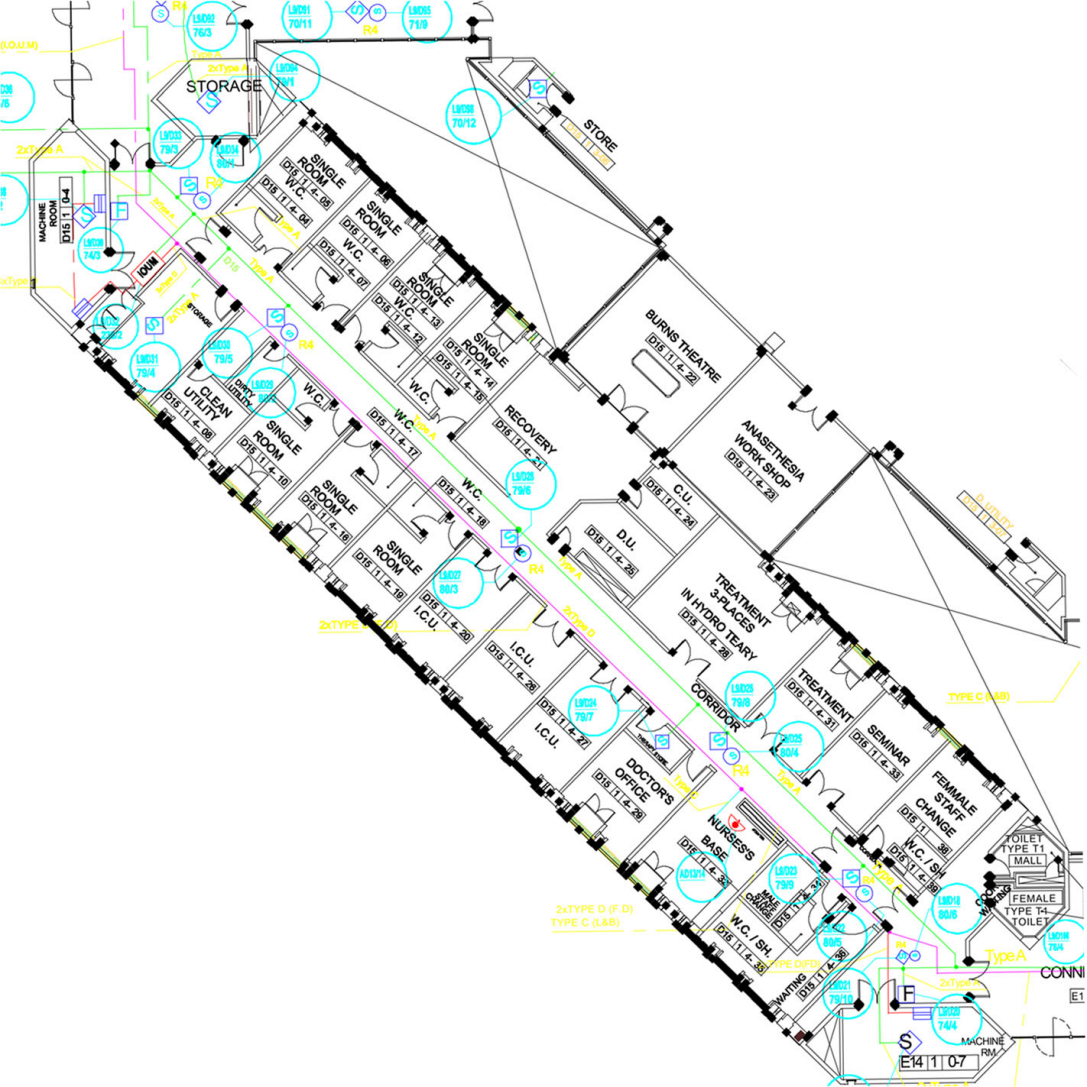
Blueprint for the burn unit that demonstrates the 10 rooms that can be adjusted to be an intensive-like care unit, operating theater, and the main entrance with changing rooms

Healthcare staff and hospital facilities cross-infections are a major concern which can affect the strongest healthcare systems in the world. The current pandemic of the COVID-19 has a significant risk of viral transmission to healthcare workers who take care of infected patients, with high reported mortality rates [7–10]. Proper management for the resources is very recommended in order to power the healthcare staff against the COVID-19 pandemic. Hill et al. adopted a new model “Corona Curtain” which can be implied for the perioperative management of urgent intubation [7]. The “Corona Curtain” described in this article represents an intuitively pragmatic, simple, innovative and cost-effective approach to attenuating the inherent risk of aerosol exposure with potential transmission of the virus to staff and providers during emergent or urgent intubations [7].

## Data Availability

Data sharing does not apply to this article as no datasets were generated or analyzed during the current study.

## Abbreviations

COVID-19: Coronavirus disease 2019
ICU: Intensive care unit
